# Magnetoencephalography Reveals Neuroprotection of COVID-19 Vaccination in Nonhuman Primates

**DOI:** 10.3390/vaccines14060543

**Published:** 2026-06-20

**Authors:** Jennifer Stapleton-Kotloski, Jared Rowland, April Davenport, Phillip Epperly, Maria Blevins, Dwayne Godwin, Daniel Ewing, Zhaodong Liang, Appavu Sundaram, Nikolai Petrovsky, Kevin Porter, John Sanders, James Daunais

**Affiliations:** 1 Department of Neurology, Wake Forest University School of Medicine, Winston-Salem, NC 27157, USA; dwayne.godwin@advocatehealth.org; 2 Department of Translational Neuroscience, Wake Forest University School of Medicine, Winston-Salem, NC 27157, USA; jared.rowland@wfusm.edu (J.R.); april.davenport@advocatehealth.org (A.D.); phillip.epperly@advocatehealth.org (P.E.); 3 Section on Infectious Diseases, Department of Internal Medicine, Wake Forest University School of Medicine, Winston-Salem, NC 27157, USA; maria.blevins@advocatehealth.org (M.B.); john.sanders@wfusm.edu (J.S.); 4 Agile Vaccines and Therapeutics Department, Defense Infectious Diseases Directorate, Naval Medical Research Command, Silver Spring, MD 20910, USA; daniel.f.ewing.civ@health.mil (D.E.); zhaodong.liang.civ@health.mil (Z.L.); appavu.k.sundaram.civ@health.mil (A.S.); 5 Vaxine Pty Ltd., Warradale, SA 5046, Australia; nikolai.petrovsky@vaxine.net; 6 Defense Infectious Diseases Directorate, Naval Medical Research Command, Silver Spring, MD 20910, USA; porter697@verizon.net

**Keywords:** magnetoencephalography, COVID-19, vaccine, neuroprotection, cynomolgus macaque, source series, synthetic aperture magnetometry

## Abstract

Background/Objectives: COVID-19, caused by the SARS-CoV-2 virus, can lead to widespread neurological and cognitive complications, even in the absence of significant structural brain abnormalities. Understanding the evolving health concerns in the context of viral infections is critical to service member readiness, fitness, and mission completion. The potential neuroprotective effects of SARS-CoV-2 vaccination remain underexplored. Methods: Using a cross-sectional, non-human primate model (female cynomolgus macaques), we employed magnetoencephalography (MEG) to assess resting-state brain activity following vaccination with escalating doses of a novel psoralen-inactivated SARS-CoV-2 vaccine (PsIV) or a combination of PsIV and a DNA vaccine (prime boost), and subsequent challenge with the Delta variant (SARS-CoV-2 B.1.617.2). MEG scans were acquired 41 days after inoculation. Source series were constructed for 42 regions of interest for each subject, and band power was computed. Results: Band power demonstrated substantial preservation of neural activity across multiple brain regions in vaccinated subjects compared to unvaccinated controls following viral challenge. Significantly lower power was observed across the brain at all bandwidths in the unvaccinated group relative to the prime boost group. As PsIV concentration increased, spectral power increased, with the prime boost group having the greatest power. Conclusions: This approach not only underscores the role of vaccination in mitigating neuropathology but also highlights the capability of MEG to detect subtle yet significant changes in brain function that may be overlooked by other imaging modalities. These findings advance our understanding of vaccine-induced neuroprotection and establish MEG as a powerful tool for monitoring brain function in the context of viral infections.

## 1. Introduction

Severe acute respiratory syndrome coronavirus 2 (SARS-CoV-2), the causative agent of COVID-19, has led to over 778 million infections and more than seven million deaths globally as of September 2025 [1]. Naval Medical Research Command, a vital part of Navy Medicine, has extensively studied COVID-19 in support of force health protection and medical readiness of U.S. Military Members. Beyond the acute phase, a significant proportion of individuals experience persistent symptoms, termed “long COVID,” which include fatigue, cognitive deficits [2,3,4,5], and neuropsychiatric disorders such as depression, PTSD, and anxiety [6,7,8,9]. Symptoms often last for months [10,11,12]. Viral-specific protein has been observed in postmortem human brain tissues [13], raising concerns over potential neuropathological consequences. Emerging evidence suggests that SARS-CoV-2 may exacerbate or initiate neurodegenerative processes [14], with many patients displaying marked neurological deficits [2,3,4,5,6], even in the absence of detectable structural abnormalities on conventional neuroimaging [15]. Several studies have demonstrated that abnormal imaging after COVID-19 infection correlates with symptomatology. For instance, thalamic and basal ganglia changes were linked to fatigue and memory issues [16], while grey matter atrophy corresponded to cognitive decline [14] and vascular lesions to acute neurological events [17,18]. A multiple regression analysis of ~112,000 participants demonstrated cognitive deficits following COVID-19 infection compared to uninfected individuals or those with unconfirmed infection, with larger deficits observed in patients with unresolved persistent symptoms and in those infected during the initial period of the COVID-19 pandemic when the wild-type Washington strain was the predominant strain [19]. EEG studies have revealed generalized slowing [20] and increases in theta and alpha frequencies in those experiencing cognitive issues such as brain fog [21].

Magnetoencephalography (MEG) is an alternative and non-invasive, clinically used neurophysiological technique that records biomagnetic fields generated by neural activity in real time [22]. MEG confers advantages over other modalities because, unlike functional MRI and PET, it is a direct measure of brain activity with sub-millisecond temporal resolution, and unlike EEG, it possesses submillimeter spatial resolution [23]. Through the use of magnetic source imaging methods (MSI) such as synthetic aperture magnetometry (SAM), biomagnetic activity can be directly mapped in brain space [24,25]. MSI can also construct virtual electrodes (source series) for any voxel in the brain, providing full bandwidth and continuous time series of activity that correspond to the local field potentials recorded with actual invasive electrodes, enabling the detection of subtle yet clinically significant changes in neural activity.

In this study, we investigated the feasibility of using MEG to identify neuroanatomical substrates underlying the cognitive deficits reported by COVID-19 patients, particularly in the context of post-acute sequelae. Additionally, we sought to determine whether vaccination against SARS-CoV-2 could confer neuroprotection against COVID-19-related alterations in brain function. Using a non-human primate model, we employed MEG to assess resting-state brain activity following vaccination with a novel psoralen-inactivated SARS-CoV-2 vaccine (PsIV) or the PsIV in combination with a DNA vaccine and subsequent challenge with the Delta variant (SARS-CoV-2 B.1.617.2). Our findings indicate that vaccination significantly mitigates COVID-19-related reductions in global resting-state brain function as measured by MEG. This suggests not only a critical neuroprotective effect of vaccination but also underscores MEG’s potential as a powerful tool for detecting and monitoring the neurological impacts of infection, offering insights that conventional imaging techniques may overlook.

## 2. Materials and Methods

### 2.1. Subjects

In this follow-on, cross-sectional study, we leveraged NHPs that were subjects in an ongoing vaccine dose escalation study [26] to assess the neuroprotective potential of vaccination to ameliorate the impact of SARS-CoV-2 infection on resting state brain function as measured by MEG.

All experiments were conducted in compliance with the Animal Welfare Act and in accordance with the *Guide for the Care and Use of Laboratory Animals* [27,28] and followed the ARRIVE reporting guidelines [29]. The study was reviewed and approved by the Wake Forest University School of Medicine Institutional Animal Care and Use Committee (IACUC) and the U.S. Navy Bureau of Medicine and Surgery (BUMED) in compliance with all applicable federal regulations governing the protection of animals and research. The IACUC and BUMED reviews included the proposed research question, key design features, and analysis plan. A total of 20 female cynomolgus macaques (Macaca Fascicularis, four to six years old and with an average weight of 5.8 kg at the beginning of the study) were subjects in this study. One of the control subjects was omitted from the study due to an incomplete MRI, which prevented MEG analysis. All data from the remaining subjects were utilized. Treatment- and infection-naïve healthy animals were screened for antibodies prior to immunization. Female monkeys were chosen because females mount better immune responses to vaccination compared to males. The animals were housed in indoor pens in groups of three to four until they were transferred to the ABSL-3 facility for challenge with live virus, at which time they were pair-housed in standard quad caging (0.75 × 1.75 × 1.80 m; Allentown Caging, Allentown, PA, USA) with removable wire-mesh partitions for separating animals when necessary. The non-human primates (NHPs) in the study were not randomly assigned to treatment groups. Instead, they were enrolled in a dose-escalation vaccine study where housing groups were sequentially assigned to different dose levels rather than by individual randomization. The technician responsible for animal care was aware of group allocation at all stages of the experiment and controlled which animals received specific vaccine doses, and animal identification was blinded to all except the technician. All other individuals were blinded to group allocation until final data analysis, at which time group allocation was revealed.

### 2.2. Preparation and Inactivation of SARS-CoV-2 PsIV and DNA Vaccines

SARS-CoV-2 PsIV was prepared as described [30]. The SARS-CoV-2 PsIV and DNA vaccines were purified and characterized as previously described [26]. Anti-SARS-CoV-2 neutralizing antibodies in serum were assayed using a microneutralization test against the SARS-CoV-2 Washington strain and the SARS-CoV-2 Delta variant as published previously [30].

### 2.3. Immunogenicity Assessment of SARS-CoV-2 PsIV Vaccine in Nonhuman Primates

The SARS-CoV-2 PsIV vaccine alone and in a prime-boost regimen using two doses of DNA vaccine followed by boosting with SARS-CoV-2 PsIV was evaluated for immunogenicity in female cynomolgus monkeys as shown in Table 1. Five groups of eight animals each were immunized by intramuscular injection (IM) with two doses of different amounts of SARS-CoV-2 PsIV with 10 mg of Advax-2 adjuvant per dose on days 0 and 30. Animals in group 1 (Control group) received Advax-2 adjuvant in PBS on days 0 and 30. Animals in group 2–4 received different amounts of SARS-CoV-2 PsIV (0.075–3.75 µg of spike protein equivalent per dose) with Advax-2 adjuvant on days 0 and 30. Animals in group 5 (prime-boost group) received a DNA vaccine encoding the full-length SARS-CoV-2 spike glycoprotein [26] on days −30 and 0, followed by a boosting dose of SARS-CoV-2 PsIV (3.75 µg spike protein equivalent) on day 30. All animals were bled on days −30, 0, 30, 37, 51, 90, and 120, and serum preparations were tested for the presence of anti-SARS-CoV-2 neutralizing antibodies using the microneutralization assays. No unexpected adverse signs of vaccination were observed. Ketoprofen was administered at the time of vaccination, and animals were observed daily until study end.

The ABSL3 environment accommodates caging for 20 monkeys at one time. On day 103, four animals from each of the groups 2–5 and four animals from the control group were moved to the ABSL-3 facility to acclimate for 7 days in preparation for a challenge with the live SARS-CoV-2 virus. On day 111, these twenty animals were challenged with 2 × 10^5^ PFU of SARS-CoV-2 Delta strain in a 0.5 mL solution, via intranasal instillation (1 × 10^5^ PFU per nostril). These groups served as subjects in the current MEG study. Blood was drawn from all animals on days 111 and 126. Nasal swabs and throat swabs were collected on alternate days starting from day 111 until day 125. Results of these assays are described here [26]. Following viral challenge, NHPs were observed daily for indications of respiratory dysfunction, fever, and depression. They were weighed, and body temperature was measured at each anesthetic event. Every other day, supplemental fluids were provided subcutaneously as needed.

### 2.4. Structural MRI

Prior to the start of the MEG study, all animals were tattooed at the nasion and preauricular positions for placement of MEG fiducials and MRI-compatible biolipid rings for subsequent T1-weighted structural MRIs. The tattoos are necessary to position the biolipid rings in the same location as the MEG fiducials to subsequently co-register the MEG and MRI data. After shaving and aseptically preparing the sites, a Spaulding Tattoo gun with a sterile tattoo needle was used for each monkey. Prior to and following tattoo application, triple antibiotic ointment was applied to the site. T1-weighted 3D MPRAGE MRIs of each subject were acquired on a 3T Siemens Skyra using a 32-channel head coil (Siemens AG, Erlangen, Germany). The MRIs were acquired after the vaccination and viral challenge occurred, at approximately 95 days post-infection.

### 2.5. Resting State MEG Recording and Analysis

All animals were fasted overnight from food but allowed access to water. On the MEG recording day, animals were sedated with ketamine and transported to the MEG suite via an IACUC-approved transport route. The animals were removed from the transport box and placed on a blanket-covered cart. An angiocatheter was placed into the saphenous vein of one leg for propofol administration. Each monkey received a bolus of propofol (2.0–4.0 mg/kg, i.v.) for intubation. Once intubated, they were maintained on propofol (~200 mg/kg/min) via syringe pump delivery for the duration of the recording procedure. The animals were covered with warming gel packs to maintain body temperature. Head localization was achieved by placing nasion and pre-auricular fiducial coils over the tattoo locations. Head motion was reduced to negligible levels (<0.2 mm) under anesthesia.

**Figure 1:**
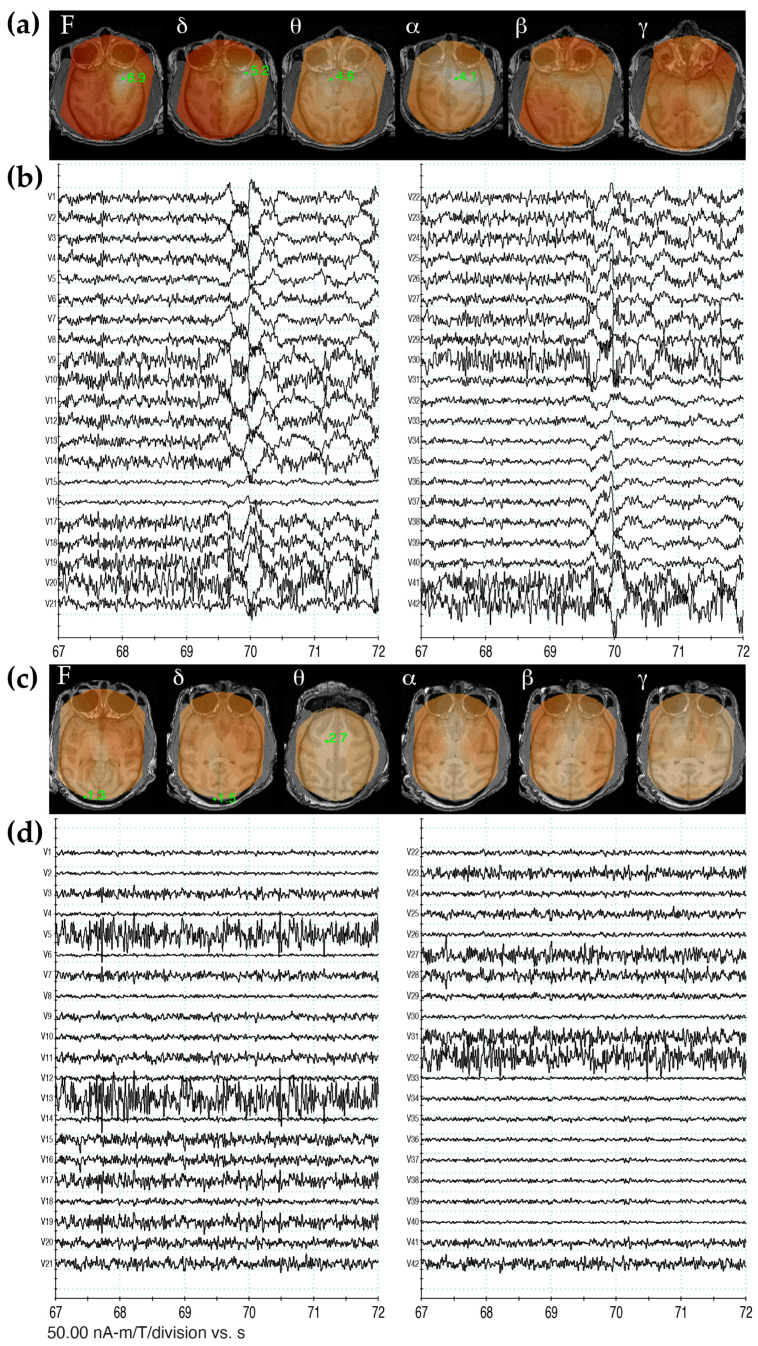
Example SAM resting state maps and MEG virtual electrodes for a vaccinated and unvaccinated subject. (**a**) Example axial slices of the SAM SPMs (voxel size = 500 μm^3^) for the full bandwidth as well as for the canonical frequency bands in a representative vaccinated subject. Green dot and number indicate a peak (local maximum) in the SPM plus the associated Ƶ-score. Peaks localize to underlying generators of brain activity. (**b**) Virtual electrode traces for the 42 ROIs (shown filtered at 1–40 Hz for viewing purposes) for a 5 s window for the vaccinated subject. (**c**) SAM maps for an unvaccinated subject. (**d**) Virtual electrodes for the unvaccinated subject. All maps are in radiological coordinates. δ, delta band; θ, theta; α, alpha; β, beta; γ, gamma; F, full bandwidth.

MEG recordings were obtained using a whole-head CTF Systems instrument equipped with 275 first-order axial gradiometer coils and 29 reference sensors housed within a magnetically shielded room (Vacuumschmelze GmbH & Co.; Hanau, Germany). Five minutes of resting state data were acquired in the supine position. Neuromagnetic responses were sampled at 2400 Hz with a bandwidth of DC-600 Hz. All preprocessing and beamforming were performed in the CTF MEG™ Software package (CTF MEG Neuro Innovations, Inc., Coquitlam, BC, Canada). Data were preprocessed using synthetic third-order gradient balancing, whole-trial DC offsetting, and band-pass filtering from DC-80 Hz with powerline filtering [23,31,32]. MEG data were co-registered with the monkey’s anatomical MRI based on the fiducials. From this, a multiple-overlapping-spheres model of the head and whole brain volume was generated [33]. Whole-brain, resting state, noise-normalized, Ƶ-deviate synthetic aperture magnetometry (SAM) [34,35] statistical parametric maps of biomagnetic activity were constructed from the MEG data on a per-subject basis at a bandwidth of DC-80 Hz and with a voxel size of 2 mm to ensure coregistration with the MRI. For display purposes (see Figure 1), two representative NHPs (one control and one prime boost subject) were also beamformed with SAM at delta (DC-4 Hz), theta (4–8 Hz), alpha (8–13 Hz), beta (13–30 Hz), gamma (30–80 Hz), and the full bandwidth (DC-80 Hz) and with a voxel size of 500 μm^3^.

**Figure 2:**
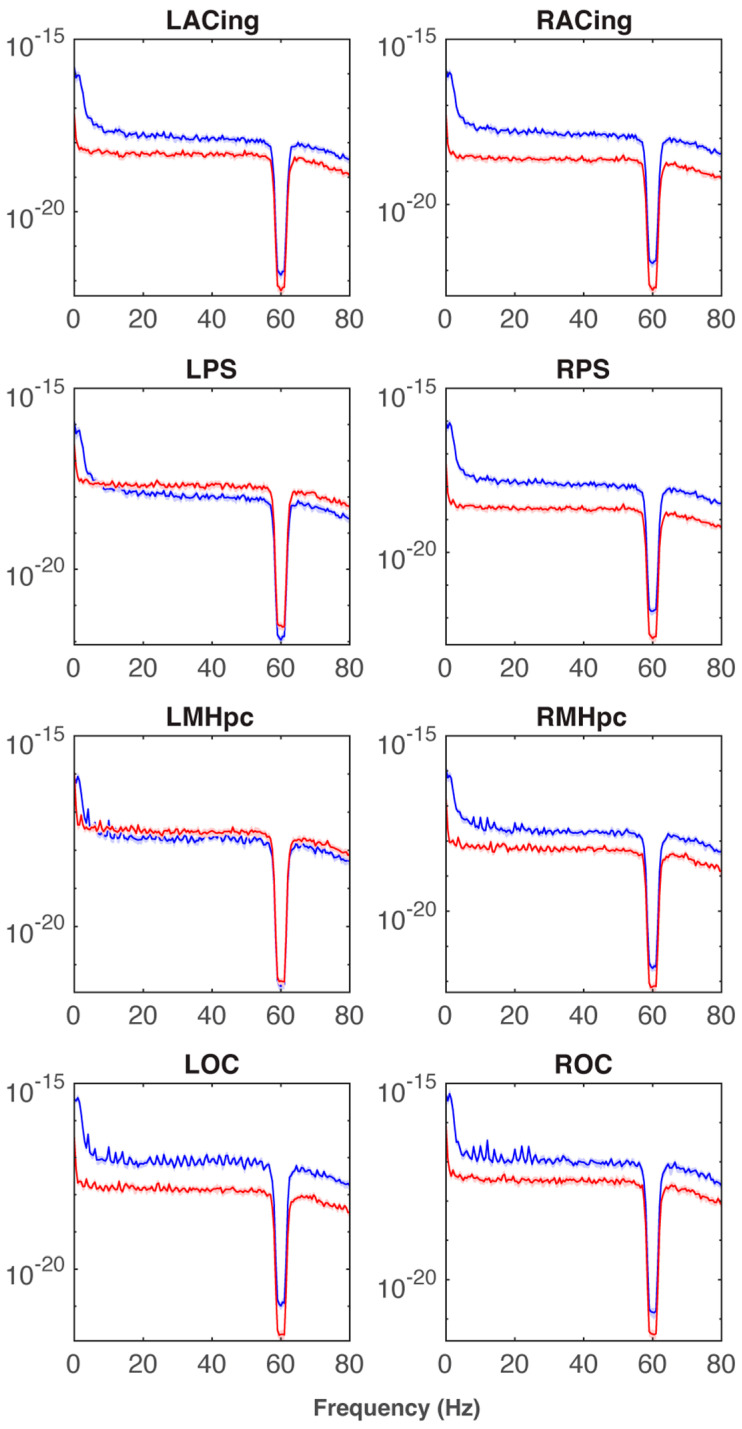
Four bilateral pairs of power spectral densities and 95% confidence intervals for select ROIs for a vaccinated (blue) and unvaccinated (red) subject. PSDs for left and right anterior cingulate (ACing), principal sulcus (PS), middle hippocampus (MHpc), and olfactory cortex (OC) virtual electrodes were calculated for a bandwidth of DC−80 Hz; MEG data were powerline filtered prior to virtual electrode construction. The y-axes are in units of T^2^/Hz.

For each animal, 42 non-adjacent bilateral regions of interest (ROIs) were manually identified in native brain space on each animal’s MRI by a primate neuroanatomist (J.D.). ROIs were chosen based on existing literature describing the impact of COVID-19 infection on brain volumetric measures [14], fluorodeoxyglucose uptake, or those areas underlying executive and cognitive function. The list of ROIs includes: L/R ACing, left/right anterior cingulate cortex; L/R MO, medial orbitofrontal cortex; L/R LO, lateral orbitofrontal cortex; L/R PS, principle sulcus; L/R NAc, nucleus accumbens; L/R Cau, caudate n.; L/R PostPut, posterior putamen; L/R precuneus; L/R Am(lat), lateral amygdala; L/R Am(BM-BL), basomedial/basolateral amygdala; L/R MHpc, middle hippocampus; L/R AHpc, anterior hippocampus; L/R CHpc, central hippocampus; L/R CbA, anterior cerebellum; L/R CbP, posterior cerebellum; midline Vermis; L/R AI, anterior insula; OB, midline olfactory bulb; L/R OB, olfactory bulb; L/R Obpost, posterior olfactory bulb; L/R Obmed, medial olfactory bulb; and L/R OC, olfactory cortex. Virtual electrodes (source series, equivalent to local field potentials, DC-80 Hz bandwidth, and five minutes in length) were extracted for each ROI, divided into 20 s, non-overlapping epochs (15 epochs per virtual electrode for each animal), and the power spectral densities (PSDs) for each epoch were computed by Welch’s method in MATLAB 2024a (Natick, MA, USA: The MathWorks Inc., RRID:SCR_001622). 95% confidence intervals (CIs) were calculated for the PSDs for Figure 2 using the mean and standard deviations of the PSDs and the critical value of the t-distribution for the appropriate sample size. For all subjects, the PSDs were split into the canonical frequency bands of delta (DC-4 Hz), theta (4–8 Hz), alpha (8–13 Hz), beta (13–30 Hz), and gamma (30–80 Hz), as well as the full bandwidth (DC-80 Hz), and total power was measured within each band.

### 2.6. Experimental Design and Statistical Analysis

For the MEG data, multiple linear regression models were constructed in R version 4.2.0, “Vigorous Calisthenics” (RRID:SCR_001905) [36], with separate models for spectral power within each of the frequency bands. Each modeled the spectral power for a given bandwidth (delta, theta, alpha, beta, gamma, or full bandwidth; pooled across subjects (*n* = 4 per group and 3 for controls) and epochs (*n* = 15)) as a function of vaccine group (groups 1–5), ROI (*n* = 42), and the interaction of vaccine group and ROI (*n* = 210). All factors were modeled as indicators, and the power for each of the bandwidths was modeled as a continuous variable. Comparisons were two-sided. Given significant main effects of vaccine group on spectral power, Tukey HSD post hoc tests were conducted within each bandwidth to determine which of the vaccine groups were significantly different from each other for a particular frequency band, with an overall family-wise error rate (FWER) of 0.05. Given the significant interaction between vaccine group and brain region, simple effects contrasts were conducted within each bandwidth to determine if power differed between the vaccine groups for each brain ROI. Simple effects contrasts were conducted with the phia package version 0.2–1 in R [37]. The Holm method was used to control the FWER at an overall alpha of 0.05 for the simple effects test; this method is equivalent to the Bonferroni method in controlling Type I error but is better at controlling the Type II error rate [38].

To assess dose-dependent effects of increasing PsIV concentration on total spectral power, a final multiple linear regression model was constructed in which the total power across the full bandwidth was modeled as a numeric, and not categorical, function of the PsIV concentration (*n* = 4), ROI (*n* = 42), and the interaction between ROI and PsIV concentration (*n* = 168). The ROI factor was modeled as an indicator variable, and the full bandwidth power was modeled as a continuous variable. The prime boost group was omitted from this analysis because it is a combination of two vaccines. Given significant main effects of PsIV vaccine concentration on spectral power, the estimated coefficient for PsIV concentration was extracted to assess the magnitude of the dose-dependent vaccine effect size on preserving brain function.

Akaike information criterion (AIC) model selection with a correction for small sample size was used to distinguish among a set of possible models describing the relationship between full bandwidth power, vaccine group, ROI, and the interaction between ROI and vaccine group using the AICcmodavg package version 2.3.3 in R [39]. Specifically, three linear models were constructed, in which power was modeled as a function of vaccine group, as a function of vaccine group and ROI, and as a full model including both terms and their interaction. The best-fit model, carrying ~100% of the cumulative model weight, included both parameters and the interaction effect. Additionally, the full model, which included both parameters and the interaction, fit the band power significantly better (*p* < 2.2 × 10^−16^) than the model containing the sum of the terms. Likewise, the model containing the sum of the parameters fit significantly better (*p* < 2.2 × 10^−16^) than the power modeled as a function of vaccine group alone. Collectively, these results support the full model as best fitting the spectral data. See Box S1 for further details.

Group size was determined using spectral power calculated from the 42 ROIs. An ANOVA was conducted in SAS Enterprise Guide version 8.3 (SAS Institute Inc., Cary, NC, USA), including main effects of group (group 1, unvaccinated, vs. group 5, prime boost) as well as time with power in the alpha band as the dependent variable. Groups were significantly different after false discovery rate correction at all but three ROIs. Power analysis was conducted using means and standard deviations from this preliminary analysis for the anterior cingulate (vaccinated mean = 1.55, SD = 1.74; unvaccinated mean = 0.26, SD = 0.26) and orbitofrontal cortex (vaccinated mean = 6.76, SD = 10.68; unvaccinated mean = 0.34, SD = 0.16). Using a range of +/− one standard deviation from the mean, statistical power for the ACC ranged from 0.907 to 0.999 and for the orbitofrontal cortex from 0.904 to 0.999. These results suggest a group size of *n* = 4 was adequately powered to determine direct contrasts between the groups.

## 3. Results

### 3.1. Immunogenicity of SARS-CoV-2 PsIV in NHPs

In this follow-on, cross-sectional study, we leveraged NHPs that were subjects in an ongoing vaccine dose escalation study [26] to determine the feasibility of applying MEG to record resting-state (RS) brain function in female cynomolgus monkeys following vaccination with a psoralen-inactivated vaccine (PsIV) or a combination of a DNA vaccine and PsIV against SARS-CoV-2 followed by challenge with the live Delta strain (SARS-CoV-2 B.1.617.2). This dose-escalation study demonstrated that immunologic response and virologic protection correlated with the administered dose, with the most significant effect observed in the heterologous prime-boost group (DNA plus PsIV) [26].

### 3.2. Neuroprotective Effects of Vaccination

The neuroprotective potential of vaccination to ameliorate the impact of SARS-CoV-2 infection on brain function was investigated in the 20 animals that underwent viral challenge. As such, four animals from each group in Table 1 were challenged with 2 × 10^5^ PFU of SARS-CoV-2 Delta strain on day 111 via intranasal instillation (1 × 10^5^ PFU per nostril). Approximately 41 days after all monkeys were inoculated with live virus, resting state (RS) MEG scans were acquired in the five groups of subjects. As this is a cross-sectional feasibility study, a single MEG scan was recorded for each subject, and a between-subjects design was employed to compare the neurological effects of vaccination following COVID-19 exposure across groups. One of the subjects from the control group was omitted from the study due to an incomplete MRI. SAM statistical parametric maps (SPMs) were constructed for the canonical frequency bands of delta (DC-4 Hz, δ), theta (4–8 Hz, θ), alpha (8–13 Hz, α), beta (13–30 Hz, β), gamma (30–80 Hz, γ), and for the full bandwidth (DC-80 Hz, F) for the remaining animals (*n* = 19). Source series (virtual electrodes) were constructed for 42 regions of interest (ROIs; see Methods for list) for each subject based on prior reports of the neurological effects of COVID-19, and power spectral densities were constructed from the source series for each ROI.

Figure 1 depicts the SAM RS SPMs for two representative animals and their associated virtual electrodes while the subjects were lightly anesthetized under propofol to demonstrate the observable effects of vaccination at an individual level for the preservation of neural activity. Panels a and b correspond to an animal from the prime-boost group (group 5), and panels c and d correspond to an unvaccinated control subject (group 1). The SAM SPMs show that the unvaccinated monkey had reduced noise-normalized neuromagnetic power at all bandwidths in comparison to the vaccinated animal after both had been exposed to SARS-CoV-2 B.1.617.2. The source series for the vaccinated subject exhibited widespread synchrony and slow oscillatory activity, such as epochs of K-complexes and delta waves, examples of which are visible at ~70 and 71 s, respectively, in Figure 1b. In contrast, the virtual electrodes for the unvaccinated subject (Figure 1d) did not appear to be widely synchronized and lacked identifiable waveforms, the absence of which is indicative of encephalopathy. Figure 2 depicts example virtual electrode power spectral densities (PSDs) and 95% confidence intervals constructed for four bilateral ROIs from the two representative subjects. Relative to the vaccinated subject (blue), several ROIs for the unvaccinated subject exhibited decreased power across the entire bandwidth.

**Figure 3:**
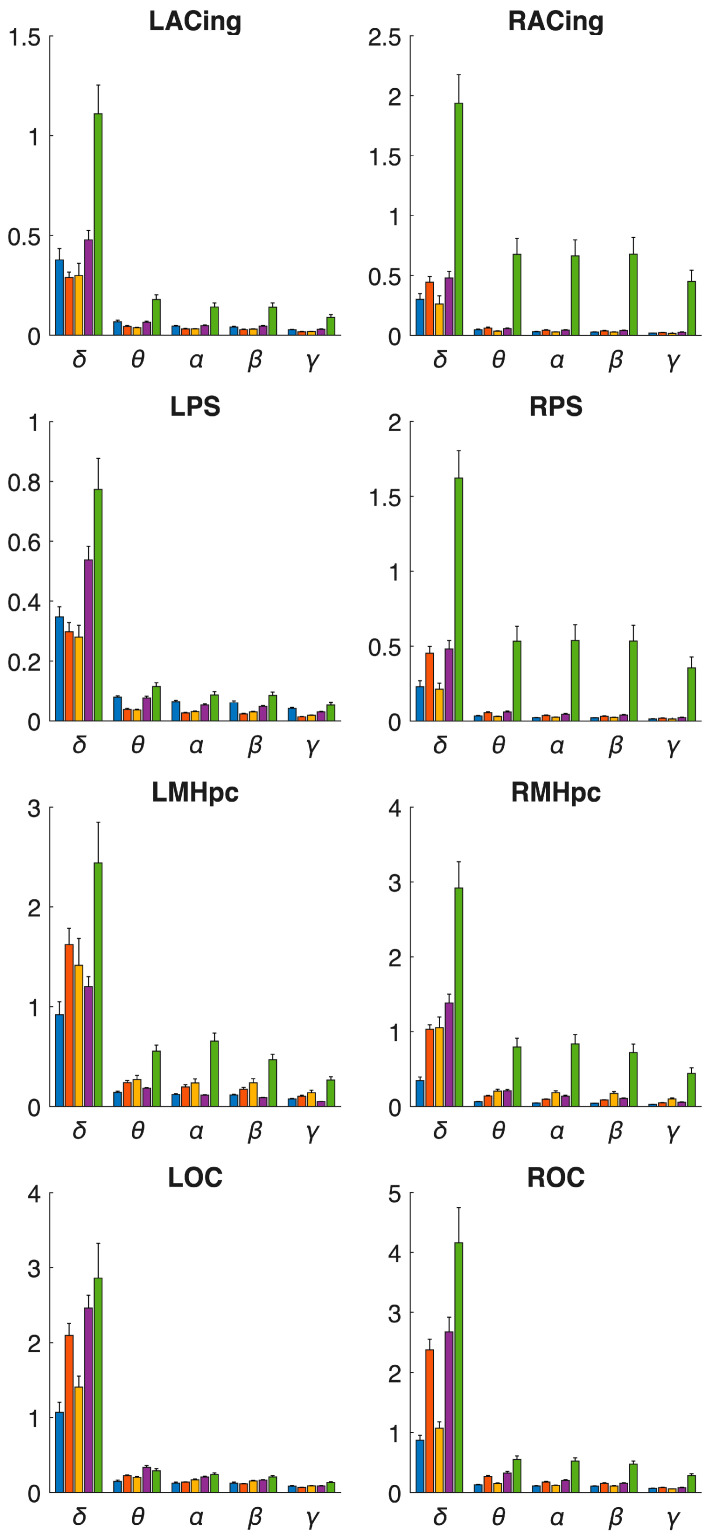
Average spectral power + SEM per frequency band for example ROIs for control and vaccine groups. Blue bars, group 1; group 2, orange; group 3, yellow; group 4, purple; group 5, green. Same ROIs as in Figure 2. The y-axes are in units of 10^−17^ T^2^.

**Figure 4:**
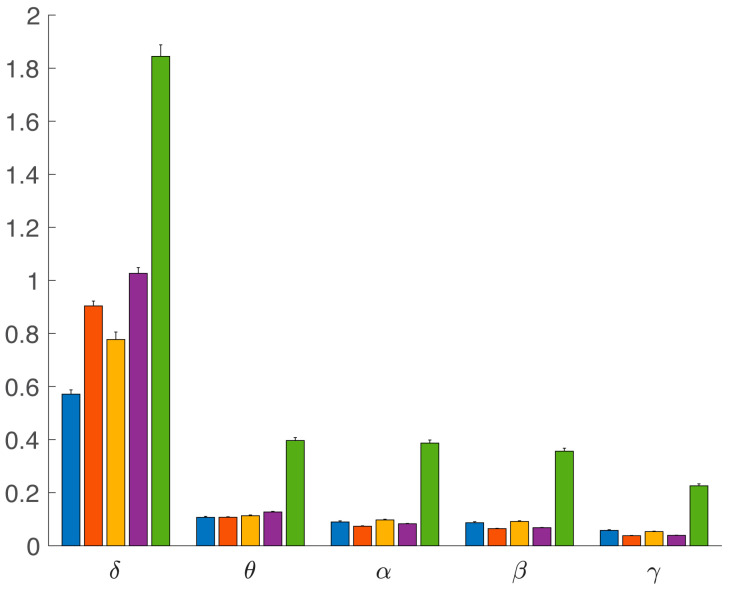
Average spectral power + SEM per frequency band pooled across all ROIs for control and vaccine groups. Blue bars, group 1; group 2, orange; group 3, yellow; group 4, purple; group 5, green. The y-axes are in units of 10^−17^ T^2^.

Figure 3 depicts the average virtual electrode power + standard error of the mean (SEM) per frequency band for the control group (15 epochs per virtual electrode for each animal; three control animals) and for each of the vaccine groups (15 epochs per virtual electrode for each animal; four animals per group) for the same representative brain areas depicted in Figure 2, and Figure 4 depicts the average spectral power + SEM per frequency band pooled across all ROIs (15 epochs per virtual electrode for each animal; 42 ROIs) for each of the groups (*n* = 4 per group with the exception of controls, where *n* = 3) to illustrate the effects of vaccination on whole brain activity. In particular, the prime-boost group exhibited consistently greater spectral power across all frequency bands for nearly all ROIs. Indeed, multiple linear regression models constructed for the spectral power at each frequency band indicated significant omnibus effects for vaccine group, ROI, and the interaction between vaccine group and ROI, with all *p*’s < 2.2 × 10^−16^. Given the significant main effects of vaccine group on spectral power, Tukey HSD post hoc tests were conducted within each bandwidth to determine which of the vaccine groups were significantly different from each other for a particular frequency band, and with an overall family-wise error rate (FWER) of 0.05. The results for these post hoc tests are presented in Table 2 and indicated that the prime boost group had consistently greater spectral power than the controls and the other vaccine groups at all frequency bands.

Given the significant interaction term, simple effects contrasts were performed with a Holm correction for the FWER such that the spectral power per bandwidth was contrasted for each of the vaccine groups at each ROI. Table 3 depicts examples of these contrasts for the four representative bilateral brain regions, and Table S1 depicts the full set of contrasts for all 42 ROIs. Significant differences (*p* < 0.05 to *p* < 2.2 × 10^−16^) in resting state band power were detected in all ROIs for the different vaccine groups following infection with the SARS-CoV-2 Delta variant. The greatest differences were detected when each of the vaccine groups was compared to either group 1 (unvaccinated) or group 5 (prime boost). Specifically, significantly lower RS power was observed across the brain at all bandwidths in the unvaccinated group relative to the prime boost group 41 days post-infection. In general, the prime boost group exhibited significantly higher power across the brain relative to the other vaccine groups. It appeared that as the PsIV concentration increased, the observed power increased, with the prime boost group having the greatest power.

To assess dose-dependent effects of PsIV concentration on total spectral power, a second multiple linear regression model was constructed in which the total power across the full bandwidth was modeled as a numeric, and not categorical, function of the PsIV concentration, ROI, and the interaction between ROI and PsIV concentration. (The prime boost group was omitted from this analysis because it is a combination of two vaccines.) There were significant omnibus effects for vaccine concentration (*p* < 0.001), ROI (*p* < 2.2 × 10^−16^), and the interaction between vaccine concentration and ROI (*p* < 2.2 × 10^−16^). Importantly, the estimated regression coefficient for the PsIV concentration was 0.81, indicating that for every 1 mg increase in PsIV concentration, the full bandwidth spectral power increased 0.81 units. However, in the original regression model of full bandwidth spectral power in which the prime boost group was included as a categorical variable, the estimated regression coefficient for the prime boost vaccine was 18.38, indicating an exceptionally strong effect on spectral power.

## 4. Discussion

COVID-19 is associated with neurobiological deficits in a large proportion of patients, many of whom exhibit abnormal neuroimaging results, including abnormal background findings in 96% of patients who receive continuous EEG [17,18,20,40,41]. This study demonstrates the feasibility of MEG as a sensitive tool for detecting neurophysiological changes following COVID-19 infection. MEG scans demonstrated substantial preservation of neural activity across brain regions in vaccinated subjects compared to unvaccinated controls following viral challenge. The results from our study are consistent with EEG findings in COVID-19 patients [42], but unlike EEG, MEG possesses submillimeter spatial resolution, enabling the localization of activity from specific cortical and subcortical brain regions [23,35,43,44].

Animal models, particularly NHPs, are an important component of pre-clinical efforts to understand the pathogenesis of SARS-CoV-2 and are critical for studying viral/host factors, disease transmission and pathogenicity, and for testing promising vaccines and antiviral drugs. Macaques have a long history as subjects in human infectious disease studies [45], most recently to investigate the neurobiological consequences of COVID-19 infection [46], and findings in macaques are likely to parallel human findings. An advantage of the NHP model is the ability to track the neurobiological consequences of COVID-19 infection from a known exposure date in a controlled environment in treatment-naïve subjects. Using this model, we identified changes early after vaccination and challenge with live virus.

Only a small number of studies have utilized MEG to record neuromagnetic activity (somatosensory or auditory) from nonhuman primates [47,48,49]. Our group has previously applied MEG to measure brain activity in multiple NHP species and treatment conditions, mapping the impacts of alcohol consumption, visual stimulation, and optogenetic brain stimulation on cortical and subcortical activity in vervet and rhesus monkeys [23,50,51]. While the NHP brain is very small in comparison to an adult human cryogenic MEG helmet, the signal-to-noise ratio can be improved through the use of axial gradiometers [52,53], low sensor noise, minimal motion [35,44], and the use of beamformers such as SAM [34,54,55], which in turn enables sub-millimeter source localization [35,43]. Our previous work has demonstrated that SAM can reliably localize optogenetically evoked signals to known sources in the cortex and hippocampus [23], and the present study extends the use of MEG in nonhuman primates to the examination of resting state brain rhythms.

As seen in Figure 1, the example prime boost vaccinated subject exhibited characteristic resting state rhythms under propofol, including widely synchronized activity, K-complexes, and delta waves, all of which are consistent with light/moderate sedation and all of which occur in humans. The visibility of such common resting-state waveforms also provides additional support for the ability of human MEG helmets, in conjunction with SAM [23,56], to detect nonhuman primate brain activity, especially given the source strength for the K-complexes of >50 nA·m. In contrast, the unvaccinated subject lacked the observable and normal electrographic architecture under propofol sedation. While individual susceptibility to anesthesia varies widely [57], this animal was unlikely to have been more deeply sedated than the vaccinated subject since all subjects in this study were maintained at the same μg/kg/min propofol concentration and the source series of the unvaccinated subject did not show signs of slowing or burst suppression. Likewise, the lack of synchrony in the unvaccinated subject’s case is unlikely due to being less sedated than the vaccinated subject because the PSD slopes do not appear to differ between the two subjects. If the unvaccinated subjects were less sedated, low frequency power should decrease, and higher frequency power should increase, changing the PSD slopes [58]. Instead, the PSD curves are shifted downward, indicating less total power across the spectrum. The lack of normal propofol sedation architecture in the source series and a full bandwidth decrease in power for the unvaccinated subject in comparison to the vaccinated subject indicate a disruption in brain function most likely consistent with encephalopathy. While COVID-19-related neuroinflammation could theoretically sensitize animals to propofol, identical weight-adjusted dosing across groups, equivalent PSD slopes, and the absence of burst suppression in the unvaccinated subject collectively argue against differential anesthetic depth as the basis for the observed MEG differences.

As a group, the unvaccinated controls exhibited the lowest spectral power, likely because of COVID-19-induced encephalopathy. As PsIV concentration increased, full bandwidth spectral power increased across the ROIs, suggesting that PsIV vaccination conferred dose-dependent neural protection of resting state rhythms. The greatest effect was evident for the heterologous prime-boost vaccination group, which exhibited the greatest spectral power across ROIs in comparison to all other vaccination groups.

Post-COVID-19 Syndrome or Long-COVID occurs in individuals with a history of SARS-CoV-2 infection in whom several symptoms last more than three months after infection, including brain fog or cognitive deficits. The COVID and Cognition (COVCOG) study reported that cognitive deficits are among the most common symptoms in Long COVID [59]. Recent evidence suggests that changes in resting state brain function underlie these deficits, and COVID-19 patients exhibit altered functional connectivity that is associated with disease severity [60,61]. Our model reflects the subacute, post-infectious stage and is not directly comparable to Long-COVID, but it does demonstrate COVID-19-related reductions in resting state brain activity 41 days after infection and, further, that vaccination protects against those deficits.

The strong relationship between neurologic protection and vaccination in an NHP model does not specifically address the pathophysiology resulting in potential encephalopathic changes. The host immune response to SARS-CoV-2 infection, including the “cytokine storm”, may be one mechanism by which COVID-19 results in neurologic and cognitive sequelae. Such T helper (TH)-1 cytokines as IL-1β, IL-6, TNF-α, and TH-2 cytokines, including IL-4 and IL-10, are elevated in serum of COVID-19 patients [62].

While evidence for direct CNS invasion of SARS-CoV-2 virus as a primary cause of neurologic symptoms remains equivocal, symptoms of COVID-19 may be attributable to neurotropic mechanisms, and these findings indicate that SARS-CoV-2 invades the CNS. Studies have detected low viral loads in brain tissue early in the infection process, though little evidence of inflammation or direct viral cytopathology was detected [13]. Rare instances of direct CNS invasion have been noted [63]. In contrast, brain lysates from COVID-19 patients who succumbed to the disease demonstrated activation of TGF-β signaling and pathways causing tau hyperphosphorylation typically associated with Alzheimer’s disease (AD) [64]. Diffuse neural inflammatory markers were found in >80% of COVID-19 patient brains, including microglia in the brainstem and hippocampus [65]. Evidence is emerging that SARS-CoV-2 proteins and/or RNA enter the brain [46,65,66,67], and SARS-CoV-2 RNA copies have been detected in the olfactory tubercle, medulla, and cerebellum and quantified by RT-qPCR [66]. A recent report suggests that SARS-CoV-2 Spike protein or its fragments may be released during infection and migrate to CNS and other tissues, resulting in cognitive dysfunction. Infusion of Spike protein into the mouse brain impacts cognitive function [68]. Additionally, RNA from two HCoV strains (229E, OC43) was detected in human brain samples collected at autopsy. Evidence for neurotropism, or affinity for neural tissue, has been documented for coronaviruses such as SARS-CoV and MERS-CoV [69]. Indeed, evidence suggests that SARS-CoV-2 spike proteins may alter the permeability of the blood-brain barrier by activating the proinflammatory response of brain endothelial cells, thus providing an avenue for CNS entry [70,71]. A recent study in NHPs demonstrated immunoreactivity to SARS-CoV-2 nucleocapsid (N) protein in the frontal lobe of rhesus macaques inoculated with SARS-CoV-2 2019-nCoV/USAWA1/2020, providing evidence of the presence of viral proteins in the brain within seven days [46]. In addition to SARS-CoV-2 N, immunolabeling for spike (Spk) markers was found in the olfactory tubercle, piriform cortex, and the olfactory pole of the entorhinal cortex, suggestive of axonal spread from nasal olfactory epithelium.

Limitations of the current study include the small group size for each vaccine dose, as well as naïve and vaccinated-unchallenged control groups. Group size was limited in the vaccine study by the capacity of the ABSL-3 facility to accommodate 20 animals, so larger samples and additional groups were not feasible here. Power calculations demonstrate that the study was adequately powered with four animals in each group (see Methods). This was borne out by the results demonstrating the robust neuroprotective potential of vaccination to ameliorate COVID-19-related encephalopathic reductions in resting state brain power. Neuropathology could manifest as hyperactivity rather than hypoactivity in some brain regions. We cannot exclude focal hyperactivity in the unvaccinated animals, and the absence of a naïve baseline precludes definitive characterization of the direction of change in all regions. Another limitation was the cross-sectional comparison of groups after animals had undergone vaccination/no vaccination and challenge with live virus. The cross-sectional nature of the study was based on the parent project that was designed to determine the efficacy of PsIV vaccination to protect against SARS-CoV-2 infection. Hence, the ability to define baseline neural activity for each subject was not possible, although the source series for the subjects could be visually inspected for the presence, alteration, or absence of expected resting state rhythms, and it is the alteration in these patterns that drives the group-wise differences in the power spectral densities. An additional limitation is that COVID-19-related neuroinflammation, via NF-κB/NLRP3 pathway activation, could theoretically modulate sensitivity to propofol, such that unvaccinated animals with active infection may have experienced differential anesthetic effects relative to vaccinated animals, representing a potential confound in the interpretation of the MEG findings. Finally, as the parent project was not terminal in nature, histology was not performed in this study, which precludes direct identification of neurodegeneration or inflammatory infiltrates.

Preliminary results demonstrate that vaccination prevents both infection-related encephalopathy and its associated decreases in resting-state spectral power. While this study evaluated vaccine platforms distinct from current FDA-approved vaccines, and our findings cannot be generalized to other COVID-19 vaccines without direct assessment, the results have broader implications. Collectively, these results not only underscore the role of vaccination in mitigating adverse neurological outcomes but also highlight the capability of MEG to detect subtle yet significant changes in brain function, even in nonhuman primates, that may be overlooked by other imaging modalities. These findings advance our understanding of vaccine-induced neuroprotection and establish MEG as a powerful tool for monitoring brain function in the context of viral infections. The demonstration of MEG’s sensitivity in detecting subtle neurological changes provides a foundation for clinical applications where this technology could be used to assess the neuroprotective effects of interventions or monitor neurological recovery in patients with post-viral cognitive symptoms, potentially offering earlier detection of neurophysiological changes than conventional imaging modalities.

## 5. Conclusions

MEG scans exhibited substantial preservation of neural activity across brain regions in a dose-dependent manner in vaccinated subjects compared to unvaccinated controls following viral challenge. This approach not only underscores the role of vaccination in mitigating neuropathology but also highlights the capability of MEG to detect subtle yet significant changes in brain function that may be overlooked by other imaging modalities. These findings advance our understanding of vaccine-induced neuroprotection and establish MEG as a powerful tool for monitoring brain function in the context of viral infections.

## Figures and Tables

**Table 1:** Vaccination schedule and dosage for SARS-CoV-2 PsIV evaluation in nonhuman primates.

Groups	Vaccine Formulation	SARS-CoV-2 PsIV Dose (Expressed as Spike Protein Equivalent) Administered
Group 1	Advax-2 + PBS (Controls) Days 0 and 30)	NA
Group 2	SARS-CoV-2 PsIV + Advax-2 (Days 0 and 30)	0.075 µg spike protein/dose
Group 3	SARS-CoV-2 PsIV + Advax-2 (Days 0 and 30)	0.750 µg spike protein/dose
Group 4	SARS-CoV-2 PsIV + Advax-2 (Days 0 and 30)	3.75 µg spike protein/dose
Group 5	Plasmid DNA (Days −30 and 0) SARS-CoV-2 PsIV + Advax-2 (Day 30)	5 mg DNA/dose, and 3.75 µg spike protein/dose

**Table 2:** Tukey HSD contrasts between vaccine groups for each bandwidth. The Groups column denotes contrasts between pairs of vaccine groups. Group 1, controls; 2, 0.075 µg PsIV; 3, 0.75 µg PsIV; 4, 3.75 µg PsIV; 5, prime boost. Entries are *p*-values, a ‘-’ denotes insignificance.

Groups	δ	θ	α	β	γ
1–2	2 × 10^−16^	-	-	-	0.0026
1–3	1 × 10^−5^	-	-	-	-
1–4	2 × 10^−16^	-	-	-	0.0053
1–5	2 × 10^−16^	2 × 10^−16^	0	2 × 10^−16^	2 × 10^−16^
2–3	0.0111	-	0.0279	0.0044	0.0202
2–4	0.0154	-	-	-	-
2–5	2 × 10^−16^	2 × 10^−16^	2 × 10^−16^	2 × 10^−16^	2 × 10^−16^
3–4	1 × 10^−9^	-	-	0.0195	0.0378
3–5	2 × 10^−16^	2 × 10^−16^	2 × 10^−16^	2 × 10^−16^	2 × 10^−16^
4–5	2 × 10^−16^	2 × 10^−16^	2 × 10^−16^	2 × 10^−16^	2 × 10^−16^

**Table 3:** Simple effects contrasts between pairs of vaccine groups at the level of example ROIs. The Groups column denotes contrasts between pairs of vaccine groups. Group 1, controls; group 2, 0.075 µg PsIV; group 3, 0.75 µg PsIV; group 4, 3.75 µg PsIV; group 5, prime boost. Entries are *p*-values, a ‘-’ denotes insignificance. ACing, anterior cingulate; PS, principal sulcus; MHpc, middle hippocampus; OC, olfactory cortex.

		Left Hemisphere					Right Hemisphere				
ROI	Groups	δ	θ	α	β	γ	δ	θ	α	β	γ
ACing	1–2	-	-	-	-	-	-	-	-	-	-
	1–3	-	-	-	-	-	-	-	-	-	-
	1–4	-	-	-	-	-	-	-	-	-	-
	1–5	0.003	0.0307	-	-	-	4 × 10^−11^	-	-	-	-
	2–3	-	-	-	-	-	-	-	-	-	-
	2–4	-	-	-	-	-	-	-	-	-	-
	2–5	0.0003	0.0048	0.0246	0.0173	0.0192	7 × 10^−11^	-	-	-	-
	3–4	-	-	-	-	-	-	-	-	-	-
	3–5	0.0004	0.0033	0.0253	0.0202	0.0214	3 × 10^−13^	-	-	-	-
	4–5	0.0057	0.0175	-	0.0433	-	2 × 10^−10^	-	-	-	-
PS	1–2	-	-	-	-	-	-	-	-	-	-
	1–3	-	-	-	-	-	-	-	-	-	-
	1–4	-	-	-	-	-	-	-	-	-	-
	1–5	-	-	-	-	-	2 × 10^−8^	2 × 10^−16^	2 × 10^−16^	2 × 10^−16^	2 × 10^−16^
	2–3	-	-	-	-	-	-	-	-	-	-
	2–4	-	-	-	-	-	-	-	-	-	-
	2–5	0.0376	-	-	-	-	3 × 10^−7^	2 × 10^−16^	2 × 10^−16^	2 × 10^−16^	2 × 10^−16^
	3–4	-	-	-	-	-	-	-	-	-	-
	3–5	0.031	-	-	-	-	7 × 10^−10^	2 × 10^−16^	2 × 10^−16^	2 × 10^−16^	2 × 10^−16^
	4–5	-	-	-	-	-	6 × 10^−7^	2 × 10^−16^	2 × 10^−16^	2 × 10^−16^	2 × 10^−16^
MHpc	1–2	0.0044	-	-	-	-	0.0055	-	-	-	-
	1–3	0.0451	0.0125	0.0266	0.0162	-	0.0042	0.0068	0.0087	0.0104	0.0282
	1–4	-	-	-	-	-	3 × 10^−5^	0.0047	-	-	-
	1–5	8 × 10^−10^	2 × 10^−15^	2 × 10^−16^	5 × 10^−12^	2 × 10^−8^	2 × 10^−16^	2 × 10^−16^	2 × 10^−16^	2 × 10^−16^	2 × 10^−16^
	2–3	-	-	-	-	-	-	-	-	-	-
	2–4	-	-	-	-	-	-	-	-	-	-
	2–5	0.0003	5 × 10^−11^	2 × 10^−16^	3 × 10^−10^	1 × 10^−7^	2 × 10^−16^	2 × 10^−16^	2 × 10^−16^	2 × 10^−16^	2 × 10^−16^
	3–4	-	-	0.0124	0.0016	0.0042	-	-	-	-	-
	3–5	7 × 10^−6^	4 × 10^−9^	2 × 10^−16^	1 × 10^−6^	4 × 10^−5^	4 × 10^−16^	2 × 10^−16^	2 × 10^−16^	2 × 10^−16^	2 × 10^−16^
	4–5	6 × 10^−8^	1 × 10^−14^	2 × 10^−16^	1 × 10^−15^	4 × 10^−12^	2 × 10^−11^	2 × 10^−16^	2 × 10^−16^	2 × 10^−16^	2 × 10^−16^
OC	1–2	3 × 10^−5^	-	-	-	-	1 × 10^−9^	0.0077	-	-	-
	1–3	-	-	-	-	-	-	-	-	-	-
	1–4	2 × 10^−8^	0.0003	-	-	-	3 × 10^−13^	0.0002	-	-	-
	1–5	5 × 10^−13^	0.0061	0.0287	-	-	2 × 10^−16^	4 × 10^−16^	6 × 10^−15^	8 × 10^−13^	2 × 10^−10^
	2–3	0.0026	-	-	-	-	1 × 10^−8^	0.0154	-	-	-
	2–4	-	0.0205	-	-	-	-	-	-	-	-
	2–5	0.0009	-	0.0367	-	0.0313	8 × 10^−15^	3 × 10^−9^	8 × 10^−13^	1 × 10^−11^	1 × 10^−10^
	3–4	4 × 10^−6^	0.0054	-	-	-	2 × 10^−12^	0.0003	-	-	-
	3–5	2 × 10^−10^	-	-	-	-	2 × 10^−16^	2 × 10^−16^	2 × 10^−16^	2 × 10^−14^	1 × 10^−12^
	4–5	-	-	-	-	-	1 × 10^−10^	2 × 10^−6^	5 × 10^−11^	2 × 10^−11^	1 × 10^−10^

## Data Availability

The raw data supporting the conclusions of this article will be made available by the authors on request.

## Supplementary Materials

The following supporting information can be downloaded at: https://www.mdpi.com/article/10.3390/vaccines14060543/s1, Table S1: Simple effects contrasts between pairs of vaccine groups at the level of each ROI; Box S1: Model Selection.

## Disclaimer

The views expressed in this article reflect the research conducted by the authors, and do not necessarily reflect the official policy or position of the Department of the Navy, the Department of Defense, or the U.S. Government. Copyright Statement: The authors are military service members or federal/contracted employees of the U.S. Government. This work was prepared as part of their official duties. Title 17 U.S.C. § 105 provides that ‘Copyright protection under this title is not available for any work of the U.S. Government.’ Title 17 U.S.C. § 101 defines a U.S. Government work as a work prepared by military service members or employees of the U.S. Government as part of that person’s official duties.

## Abbreviations

The following abbreviations are used in this manuscript:

ACing	Anterior cingulate cortex
AHpc	Anterior hippocampus
AI	Anterior insula
AIC	Akaike information criterion
Am(BM-BL)	Basomedial/basolateral amygdala
Am(lat)	Lateral amygdala
BUMED	U.S. Navy Bureau of Medicine and Surgery
Cau	Caudate n.
CbA	Anterior cerebellum
CbP	Posterior cerebellum
CHpc	Central hippocampus
CI	Confidence interval
FWER	Family-wise error rate
IACUC	Institutional Animal Care and Use Committee
IM	Intramuscular injection
L/R	Left/right
LO	Lateral orbitofrontal cortex
MEG	Magnetoencephalography
MHpc	Middle hippocampus
MO	Medial orbitofrontal cortex
MSI	Magnetic source imaging
NAc	Nucleus accumbens
NHP	Nonhuman primate
OB	Olfactory bulb
Obmed	Medial olfactory bulb
Obpost	Posterior olfactory bulb
OC	Olfactory cortex
PFU	Plaque forming unit
PostPut	Posterior putamen
PS	Principle sulcus
PSD	Power spectral density
PsIV	Psoralen-inactivated vaccine
ROI	Region of interest
RS	Resting state
SAM	Synthetic aperture magnetometry
SPM	Statistical parametric map
