# Supplementary material for: Magnetoencephalography Reveals Neuroprotection of COVID-19 Vaccination in Nonhuman Primates
Source: Vaccines (Basel). 2026 Jun 20;14(6):543. doi: 10.3390/vaccines14060543 (PMC13307603; doi:10.3390/vaccines14060543)
Supplement: Supplementary file 1 [file vaccines-14-00543-s001.zip › vaccines-4371727-supplementary.pdf]

Supplementary Material

Table S1. Simple effects contrasts between pairs of vaccine groups at the level of each ROI. Group 1, controls; group 2, 0.075 µg PsIV; group 3, 0.75 µg PsIV; group 4, 3.75 µg PsIV; group 5, prime boost. Entries are P-values, a '-' denotes insignificance. See Materials and Methods for ROI descriptions.

		Left Hemisphere						Right Hemisphere					
**ROI**	**Groups**	**δ**	**θ**	**α**	**β**	**γ**	**F**	**δ**	**θ**	**α**	**β**	**γ**	**F**
ACing	1-2	-	-	-	-	-	-	-	-	-	-	-	-
	1-3	-	-	-	-	-	-	-	-	-	-	-	-
	1-4	-	-	-	-	-	-	-	-	-	-	-	-
	1-5	0.003	0.031	-	-	-	0.012	$4×10^{-11}$	$2×10^{-16}$	$2×10^{-16}$	$2×10^{-16}$	$2×10^{-16}$	$2×10^{-16}$
	2-3	-	-	-	-	-	-	-	-	-	-	-	-
	2-4	-	-	-	-	-	-	-	-	-	-	-	-
	2-5	$3×10^{-4}$	0.005	0.025	0.017	0.019	0.002	$7×10^{-11}$	$2×10^{-16}$	$2×10^{-16}$	$2×10^{-16}$	$2×10^{-16}$	$2×10^{-16}$
	3-4	-	-	-	-	-	-	-	-	-	-	-	-
	3-5	$4×10^{-4}$	0.003	0.025	0.02	0.021	0.002	$3×10^{-13}$	$2×10^{-16}$	$2×10^{-16}$	$2×10^{-16}$	$2×10^{-16}$	$2×10^{-16}$
	4-5	0.006	0.018	-	0.043	-	0.012	$2×10^{-10}$	$2×10^{-16}$	$2×10^{-16}$	$2×10^{-16}$	$2×10^{-16}$	$2×10^{-16}$
MO	1-2	-	-	-	-	-	-	-	-	-	-	-	-
	1-3	-	-	-	-	-	-	-	-	-	-	-	-
	1-4	-	-	-	-	-	-	-	-	-	-	-	-
	1-5	-	-	-	-	-	-	$6×10^{-11}$	$2×10^{-16}$	$2×10^{-16}$	$2×10^{-16}$	$2×10^{-16}$	$2×10^{-16}$
	2-3	-	-	-	-	-	-	-	-	-	-	-	-
	2-4	-	-	-	-	-	-	-	-	-	-	-	-
	2-5	-	-	-	-	-	0.048	$4×10^{-7}$	$2×10^{-16}$	$2×10^{-16}$	$2×10^{-16}$	$2×10^{-16}$	$2×10^{-16}$
	3-4	0.025	-	-	-	-	-	-	-	-	-	-	-
	3-5	-	0.034	-	-	-	0.04	$1×10^{-11}$	$2×10^{-16}$	$2×10^{-16}$	$2×10^{-16}$	$2×10^{-16}$	$2×10^{-16}$
	4-5	-	-	-	-	-	-	$2×10^{-7}$	$2×10^{-16}$	$2×10^{-16}$	$2×10^{-16}$	$2×10^{-16}$	$2×10^{-16}$
LO	1-2	-	$5×10^{-8}$	$5×10^{-8}$	$6×10^{-9}$	$9×10^{-10}$	$3×10^{-8}$	-	-	-	-	-	-
	1-3	0.042	$7×10^{-8}$	$1×10^{-7}$	$2×10^{-8}$	$3×10^{-9}$	$5×10^{-8}$	-	-	-	-	-	-
	1-4	-	$1×10^{-5}$	$1×10^{-6}$	$2×10^{-7}$	$2×10^{-8}$	$3×10^{-6}$	-	-	-	-	-	-
	1-5	-	$8×10^{-6}$	$6×10^{-6}$	$2×10^{-6}$	$3×10^{-7}$	$1×10^{-5}$	$5×10^{-6}$	$1×10^{-13}$	$4×10^{-15}$	$2×10^{-15}$	$7×10^{-16}$	$5×10^{-16}$
	2-3	-	-	-	-	-	-	-	-	-	-	-	-
	2-4	-	-	-	-	-	-	-	-	-	-	-	-
	2-5	-	-	-	-	-	-	0.001	$3×10^{-13}$	$5×10^{-16}$	$2×10^{-16}$	$2×10^{-16}$	$1×10^{-15}$
	3-4	-	-	-	-	-	-	-	-	-	-	-	-
	3-5	-	-	-	-	-	-	$9×10^{-7}$	$9×10^{-16}$	$2×10^{-16}$	$2×10^{-16}$	$2×10^{-16}$	$2×10^{-16}$
	4-5	-	-	-	-	-	-	0.001	$1×10^{-12}$	$4×10^{-15}$	$8×10^{-16}$	$2×10^{-16}$	$6×10^{-15}$
PS	1-2	-	-	-	-	-	-	-	-	-	-	-	-
	1-3	-	-	-	-	-	-	-	-	-	-	-	-
	1-4	-	-	-	-	-	-	-	-	-	-	-	-
	1-5	-	-	-	-	-	-	$2×10^{-8}$	$2×10^{-16}$	$2×10^{-16}$	$2×10^{-16}$	$2×10^{-16}$	$2×10^{-16}$
	2-3	-	-	-	-	-	-	-	-	-	-	-	-
	2-4	-	-	-	-	-	-	-	-	-	-	-	-
	2-5	0.038	-	-	-	-	-	$3×10^{-7}$	$2×10^{-16}$	$2×10^{-16}$	$2×10^{-16}$	$2×10^{-16}$	$2×10^{-16}$
	3-4	-	-	-	-	-	-	-	-	-	-	-	-
	3-5	0.031	-	-	-	-	-	$7×10^{-10}$	$2×10^{-16}$	$2×10^{-16}$	$2×10^{-16}$	$2×10^{-16}$	$2×10^{-16}$
	4-5	-	-	-	-	-	-	$6×10^{-7}$	$2×10^{-16}$	$2×10^{-16}$	$2×10^{-16}$	$2×10^{-16}$	$2×10^{-16}$
NAc	1-2	0.005	-	-	-	-	-	$5×10^{-4}$	-	-	-	-	-
	1-3	0.015	-	-	-	-	-	-	-	-	-	-	-
	1-4	$1×10^{-4}$	-	-	-	-	-	$5×10^{-5}$	-	-	-	-	-
	1-5	$3×10^{-9}$	0.04	0.017	0.009	0.009	$8×10^{-5}$	$4×10^{-12}$	$3×10^{-15}$	$2×10^{-15}$	$2×10^{-16}$	$2×10^{-16}$	$2×10^{-16}$
	2-3	-	-	-	-	-	-	0.023	-	-	-	-	-

	2-4	-	-	-	-	-	-	-	-	-	-	-	-
	2-5	9×10^{-4}	0.029	0.026	0.012	0.011	0.002	2×10^{-4}	8×10^{-16}	4×10^{-15}	2×10^{-16}	2×10^{-16}	2×10^{-16}
	3-4	-	-	-	-	-	-	0.003	-	-	-	-	-
	3-5	2×10^{-4}	0.013	0.035	0.022	0.01	0.001	2×10^{-9}	2×10^{-16}	8×10^{-15}	2×10^{-16}	2×10^{-16}	2×10^{-16}
	4-5	0.026	0.01	0.009	0.005	0.007	0.003	0.002	1×10^{-15}	2×10^{-15}	2×10^{-16}	2×10^{-16}	2×10^{-16}
Cau	1-2	-	-	-	-	-	-	-	-	-	-	-	-
	1-3	-	-	-	-	-	-	-	-	-	-	-	-
	1-4	-	-	-	-	-	-	-	-	-	-	-	-
	1-5	0.006	0.016	0.009	0.008	0.012	0.003	5×10^{-10}	2×10^{-16}	2×10^{-16}	2×10^{-16}	2×10^{-16}	2×10^{-16}
	2-3	-	-	-	-	-	-	-	-	-	-	-	-
	2-4	-	-	-	-	-	-	-	-	-	-	-	-
	2-5	4×10^{-4}	8×10^{-6}	6×10^{-5}	8×10^{-5}	1×10^{-4}	1×10^{-5}	2×10^{-8}	2×10^{-16}	2×10^{-16}	2×10^{-16}	2×10^{-16}	2×10^{-16}
	3-4	-	-	-	-	-	-	-	-	-	-	-	-
	3-5	5×10^{-5}	3×10^{-6}	4×10^{-5}	6×10^{-5}	5×10^{-5}	3×10^{-6}	7×10^{-13}	2×10^{-16}	2×10^{-16}	2×10^{-16}	2×10^{-16}	2×10^{-16}
	4-5	4×10^{-4}	5×10^{-6}	3×10^{-5}	3×10^{-5}	5×10^{-5}	5×10^{-6}	2×10^{-9}	2×10^{-16}	2×10^{-16}	2×10^{-16}	2×10^{-16}	2×10^{-16}
PostPut	1-2	-	1×10^{-15}	2×10^{-16}	2×10^{-16}	2×10^{-16}	2×10^{-15}	-	-	-	-	-	-
	1-3	-	5×10^{-15}	5×10^{-16}	2×10^{-16}	2×10^{-16}	9×10^{-15}	-	-	-	-	-	-
	1-4	-	1×10^{-14}	2×10^{-16}	2×10^{-16}	2×10^{-16}	2×10^{-15}	-	-	-	-	-	-
	1-5	-	2×10^{-7}	3×10^{-9}	4×10^{-10}	6×10^{-12}	1×10^{-6}	4×10^{-12}	2×10^{-16}	2×10^{-16}	2×10^{-16}	2×10^{-16}	2×10^{-16}
	2-3	-	-	-	-	-	-	-	-	-	-	-	-
	2-4	-	-	-	-	-	-	-	-	-	-	-	-
	2-5	3×10^{-4}	0.003	0.006	0.007	0.01	9×10^{-4}	1×10^{-8}	2×10^{-16}	2×10^{-16}	2×10^{-16}	2×10^{-16}	2×10^{-16}
	3-4	-	-	-	-	-	-	-	-	-	-	-	-
	3-5	8×10^{-5}	0.005	0.019	0.027	0.031	0.002	2×10^{-11}	2×10^{-16}	2×10^{-16}	2×10^{-16}	2×10^{-16}	2×10^{-16}
	4-5	5×10^{-4}	0.008	0.008	0.008	0.009	0.001	5×10^{-8}	2×10^{-16}	2×10^{-16}	2×10^{-16}	2×10^{-16}	2×10^{-16}
Precuneus	1-2	-	-	-	-	-	-	-	-	-	-	-	-
	1-3	-	-	-	-	-	-	-	-	-	-	-	-
	1-4	-	-	-	-	-	-	-	-	-	-	-	-
	1-5	-	-	-	-	-	-	0.049	0.018	0.018	-	-	0.027
	2-3	-	-	-	-	-	-	0.019	-	-	-	-	-
	2-4	-	-	-	-	-	-	-	-	-	-	-	-
	2-5	0.027	0.004	0.004	0.014	0.016	0.006	0.005	5×10^{-4}	6×10^{-4}	0.003	0.004	0.0007
	3-4	-	-	-	-	-	-	-	-	-	-	-	-
	3-5	-	-	-	-	-	-	-	0.038	0.033	-	-	-
	4-5	-	0.008	0.006	0.016	0.018	0.01	0.027	0.001	0.001	0.005	0.005	0.002
Am(lat)	1-2	0.009	-	-	-	-	-	0.002	-	-	-	-	-
	1-3	0.031	-	-	-	-	-	-	-	-	-	-	-
	1-4	0.018	-	-	-	-	-	8×10^{-6}	0.004	-	-	-	0.0262
	1-5	1×10^{-5}	0.004	0.005	-	-	0.005	4×10^{-13}	2×10^{-16}	2×10^{-16}	2×10^{-16}	2×10^{-16}	2×10^{-16}
	2-3	-	-	-	0.048	-	-	0.007	-	-	-	-	-
	2-4	-	-	-	-	-	-	-	-	-	-	-	-
	2-5	-	0.003	2×10^{-4}	0.002	0.004	0.002	5×10^{-6}	2×10^{-16}	2×10^{-16}	2×10^{-16}	2×10^{-16}	2×10^{-16}
	3-4	-	-	-	-	-	-	2×10^{-5}	0.012	-	-	-	-
	3-5	0.016	-	-	-	-	-	5×10^{-13}	2×10^{-16}	2×10^{-16}	2×10^{-16}	2×10^{-16}	2×10^{-16}
	4-5	0.03	0.038	5×10^{-4}	0.003	0.002	0.001	0.003	2×10^{-11}	2×10^{-16}	2×10^{-16}	2×10^{-16}	2×10^{-16}
Am(BM-BL)	1-2	3×10^{-5}	-	-	-	-	-	7×10^{-4}	-	-	-	-	-
	1-3	-	-	-	-	-	-	-	-	-	-	-	-
	1-4	4×10^{-4}	0.031	-	-	-	-	1×10^{-11}	7×10^{-5}	-	-	-	0.0033
	1-5	1×10^{-6}	0.003	0.004	-	-	0.003	2×10^{-16}	2×10^{-16}	2×10^{-16}	2×10^{-16}	2×10^{-16}	2×10^{-16}
	2-3	1×10^{-4}	-	-	-	-	-	0.001	-	-	-	-	-
	2-4	-	-	-	-	-	-	3×10^{-4}	0.01	-	-	-	-
	2-5	-	0.043	0.001	0.009	0.009	0.014	2×10^{-16}	2×10^{-16}	2×10^{-16}	2×10^{-16}	2×10^{-16}	2×10^{-16}
	3-4	0.001	-	-	-	-	-	5×10^{-12}	3×10^{-4}	-	-	-	0.0042
	3-5	4×10^{-6}	0.009	0.006	0.048	0.027	0.001	2×10^{-16}	2×10^{-16}	2×10^{-16}	2×10^{-16}	2×10^{-16}	2×10^{-16}

	4-5	-	-	0.005	0.011	0.004	0.008	3×10^{-10}	6×10^{-14}	2×10^{-16}	2×10^{-16}	2×10^{-16}	2×10^{-16}
MHpc	1-2	0.004	-	-	-	-	-	0.006	-	-	-	-	-
	1-3	0.045	0.013	0.027	0.016	-	0.017	0.004	0.007	0.009	0.01	0.028	0.0041
	1-4	-	-	-	-	-	-	3×10^{-5}	0.005	-	-	-	0.0226
	1-5	8×10^{-10}	2×10^{-15}	2×10^{-16}	5×10^{-12}	2×10^{-8}	1×10^{-13}	2×10^{-16}	2×10^{-16}	2×10^{-16}	2×10^{-16}	2×10^{-16}	2×10^{-16}
	2-3	-	-	-	-	-	-	-	-	-	-	-	-
	2-4	-	-	-	-	-	-	-	-	-	-	-	-
	2-5	3×10^{-4}	5×10^{-11}	2×10^{-16}	3×10^{-10}	1×10^{-7}	6×10^{-10}	2×10^{-16}	2×10^{-16}	2×10^{-16}	2×10^{-16}	2×10^{-16}	2×10^{-16}
	3-4	-	-	0.012	0.002	0.004	0.008	-	-	-	-	-	-
	3-5	7×10^{-6}	4×10^{-9}	2×10^{-16}	1×10^{-6}	4×10^{-5}	5×10^{-8}	4×10^{-16}	2×10^{-16}	2×10^{-16}	2×10^{-16}	2×10^{-16}	2×10^{-16}
	4-5	6×10^{-8}	1×10^{-14}	2×10^{-16}	1×10^{-15}	4×10^{-12}	4×10^{-16}	2×10^{-11}	2×10^{-16}	2×10^{-16}	2×10^{-16}	2×10^{-16}	2×10^{-16}
AHpc	1-2	0.007	-	-	-	-	-	0.01	-	-	-	-	-
	1-3	0.005	9×10^{-4}	0.004	0.003	0.017	0.002	0.049	-	-	-	-	0.0405
	1-4	-	-	-	-	-	-	5×10^{-6}	0.002	-	-	-	0.0144
	1-5	1×10^{-9}	2×10^{-11}	2×10^{-16}	1×10^{-8}	5×10^{-6}	2×10^{-10}	2×10^{-16}	2×10^{-16}	2×10^{-16}	2×10^{-16}	2×10^{-16}	2×10^{-16}
	2-3	-	-	0.029	0.007	0.017	0.037	-	-	-	-	-	-
	2-4	-	-	-	-	-	-	0.029	0.036	-	-	-	-
	2-5	3×10^{-4}	3×10^{-8}	2×10^{-16}	2×10^{-8}	2×10^{-6}	2×10^{-8}	3×10^{-12}	2×10^{-16}	2×10^{-16}	2×10^{-16}	2×10^{-16}	2×10^{-16}
	3-4	-	0.021	0.002	2×10^{-4}	7×10^{-4}	0.001	0.005	-	-	-	-	-
	3-5	5×10^{-4}	3×10^{-4}	1×10^{-9}	0.004	0.019	4×10^{-4}	2×10^{-14}	2×10^{-16}	2×10^{-16}	2×10^{-16}	2×10^{-16}	2×10^{-16}
	4-5	1×10^{-6}	3×10^{-9}	2×10^{-16}	3×10^{-11}	9×10^{-9}	9×10^{-12}	1×10^{-6}	2×10^{-16}	2×10^{-16}	2×10^{-16}	2×10^{-16}	2×10^{-16}
CHpc	1-2	0.001	0.032	-	-	-	0.024	0.021	-	-	-	-	-
	1-3	0.024	0.016	0.026	0.019	0.049	0.013	0.002	0.003	0.003	0.007	0.017	0.0019
	1-4	-	-	-	-	-	-	0.002	0.025	-	-	-	-
	1-5	7×10^{-14}	2×10^{-16}	2×10^{-16}	5×10^{-14}	4×10^{-10}	2×10^{-16}	1×10^{-11}	1×10^{-8}	1×10^{-8}	1×10^{-6}	2×10^{-5}	2×10^{-9}
	2-3	-	-	-	-	-	-	-	-	0.025	0.038	-	-
	2-4	0.002	-	0.042	0.043	-	0.01	-	-	-	-	-	-
	2-5	3×10^{-6}	5×10^{-14}	2×10^{-16}	2×10^{-10}	1×10^{-7}	6×10^{-11}	1×10^{-6}	2×10^{-6}	2×10^{-7}	1×10^{-5}	8×10^{-5}	5×10^{-7}
	3-4	0.034	0.049	0.016	0.005	0.01	0.005	-	-	-	-	-	-
	3-5	2×10^{-8}	4×10^{-13}	2×10^{-16}	2×10^{-8}	4×10^{-6}	3×10^{-10}	7×10^{-5}	0.003	0.003	0.022	0.042	0.0018
	4-5	8×10^{-15}	2×10^{-16}	2×10^{-16}	2×10^{-16}	7×10^{-13}	2×10^{-16}	1×10^{-4}	2×10^{-4}	2×10^{-6}	3×10^{-5}	1×10^{-4}	5×10^{-6}
CbA	1-2	-	-	-	-	-	-	-	-	-	-	-	-
	1-3	-	-	-	-	-	-	-	-	-	-	-	-
	1-4	-	-	-	-	-	-	0.02	-	-	-	-	-
	1-5	2×10^{-4}	2×10^{-4}	9×10^{-4}	0.004	0.011	5×10^{-4}	2×10^{-16}	2×10^{-16}	3×10^{-13}	3×10^{-10}	5×10^{-10}	8×10^{-16}
	2-3	-	-	-	-	-	-	-	-	-	-	-	-
	2-4	-	-	-	-	-	-	0.028	-	-	-	-	-
	2-5	1×10^{-5}	4×10^{-8}	2×10^{-7}	1×10^{-6}	4×10^{-6}	7×10^{-8}	2×10^{-16}	2×10^{-16}	2×10^{-16}	8×10^{-15}	5×10^{-15}	2×10^{-16}
	3-4	-	-	-	-	-	-	-	-	-	-	-	-
	3-5	2×10^{-5}	2×10^{-7}	3×10^{-6}	1×10^{-5}	2×10^{-5}	6×10^{-7}	7×10^{-16}	2×10^{-16}	4×10^{-14}	3×10^{-11}	1×10^{-11}	2×10^{-16}
	4-5	0.009	5×10^{-7}	9×10^{-7}	3×10^{-6}	1×10^{-5}	3×10^{-6}	2×10^{-11}	2×10^{-16}	2×10^{-16}	1×10^{-13}	6×10^{-14}	2×10^{-16}
CbP	1-2	-	-	-	-	-	-	-	-	-	-	-	-
	1-3	-	-	-	-	-	-	0.008	0.036	-	-	-	0.0338
	1-4	-	-	-	-	-	-	0.029	-	-	-	-	-
	1-5	9×10^{-8}	7×10^{-7}	9×10^{-6}	2×10^{-5}	1×10^{-4}	3×10^{-7}	2×10^{-16}	2×10^{-11}	3×10^{-8}	8×10^{-7}	1×10^{-5}	3×10^{-12}
	2-3	-	-	-	-	-	-	-	-	-	-	-	-
	2-4	-	-	-	-	-	-	-	-	-	-	-	-
	2-5	2×10^{-6}	3×10^{-6}	6×10^{-6}	2×10^{-5}	6×10^{-5}	7×10^{-7}	2×10^{-16}	3×10^{-10}	3×10^{-8}	8×10^{-7}	1×10^{-5}	3×10^{-11}
	3-4	-	-	-	-	-	-	-	-	-	-	-	-
	3-5	1×10^{-8}	2×10^{-7}	2×10^{-6}	6×10^{-6}	2×10^{-5}	5×10^{-8}	3×10^{-14}	7×10^{-7}	7×10^{-5}	3×10^{-4}	0.002	1×10^{-7}
	4-5	1×10^{-5}	2×10^{-6}	3×10^{-6}	5×10^{-6}	2×10^{-5}	4×10^{-7}	6×10^{-16}	8×10^{-10}	6×10^{-8}	8×10^{-7}	9×10^{-6}	8×10^{-11}
AI	1-2	-	1×10^{-4}	4×10^{-5}	1×10^{-5}	2×10^{-6}	1×10^{-4}	0.004	-	-	-	-	-
	1-3	-	0.018	0.008	0.004	8×10^{-4}	0.012	-	-	-	-	-	-
	1-4	-	1×10^{-4}	2×10^{-5}	3×10^{-6}	4×10^{-7}	2×10^{-5}	0.042	-	-	-	-	-

	1-5	-	-	-	0.015	0.004	0.038	7×10^{-6}	2×10^{-15}	3×10^{-16}	2×10^{-16}	2×10^{-16}	2×10^{-16}
	2-3	-	-	-	-	-	-	0.01	-	-	-	-	-
	2-4	-	-	-	-	-	-	-	-	-	-	-	-
	2-5	-	0.018	0.014	0.031	0.046	-	-	3×10^{-12}	1×10^{-15}	2×10^{-16}	2×10^{-16}	1×10^{-13}
	3-4	-	-	-	0.048	-	-	-	-	-	-	-	-
	3-5	-	-	-	-	-	-	2×10^{-5}	3×10^{-16}	2×10^{-16}	2×10^{-16}	2×10^{-16}	2×10^{-16}
	4-5	-	0.016	0.007	0.015	0.018	0.015	0.008	2×10^{-12}	2×10^{-15}	2×10^{-16}	2×10^{-16}	8×10^{-15}
OB	1-2	-	-	-	-	-	-	-	-	-	-	-	-
	1-3	-	-	-	-	-	-	0.049	-	-	-	-	-
	1-4	-	-	-	-	-	-	-	-	-	-	-	-
	1-5	0.031	1×10^{-4}	5×10^{-4}	7×10^{-5}	8×10^{-5}	8×10^{-5}	0.02	1×10^{-5}	7×10^{-5}	8×10^{-6}	8×10^{-6}	9×10^{-6}
	2-3	-	-	-	-	-	-	-	-	-	-	-	-
	2-4	-	-	-	-	-	-	-	-	-	-	-	-
	2-5	-	1×10^{-4}	3×10^{-4}	2×10^{-5}	2×10^{-5}	5×10^{-5}	0.036	1×10^{-5}	3×10^{-5}	2×10^{-6}	1×10^{-6}	4×10^{-6}
	3-4	-	-	-	-	-	-	-	-	-	-	-	-
	3-5	-	0.001	0.004	6×10^{-4}	6×10^{-4}	0.004	-	2×10^{-4}	8×10^{-4}	9×10^{-5}	7×10^{-5}	0.0007
	4-5	-	3×10^{-4}	5×10^{-4}	5×10^{-5}	5×10^{-5}	1×10^{-4}	0.037	2×10^{-5}	4×10^{-5}	3×10^{-6}	2×10^{-6}	6×10^{-6}
Obpost	1-2	-	-	-	-	-	-	-	-	-	-	-	-
	1-3	-	-	-	-	-	-	-	-	-	-	-	-
	1-4	0.031	-	-	-	-	-	0.029	-	-	-	-	-
	1-5	3×10^{-4}	2×10^{-8}	4×10^{-7}	7×10^{-9}	2×10^{-8}	5×10^{-9}	1×10^{-6}	2×10^{-16}	2×10^{-16}	2×10^{-16}	2×10^{-16}	2×10^{-16}
	2-3	-	-	-	-	-	-	-	-	-	-	-	-
	2-4	-	-	-	-	-	-	-	-	-	-	-	-
	2-5	9×10^{-4}	2×10^{-8}	2×10^{-7}	1×10^{-9}	1×10^{-9}	2×10^{-9}	2×10^{-6}	2×10^{-16}	2×10^{-16}	2×10^{-16}	2×10^{-16}	2×10^{-16}
	3-4	-	-	-	-	-	-	-	-	-	-	-	-
	3-5	0.005	7×10^{-9}	6×10^{-7}	7×10^{-9}	9×10^{-9}	2×10^{-8}	4×10^{-6}	2×10^{-16}	2×10^{-16}	2×10^{-16}	2×10^{-16}	2×10^{-16}
	4-5	-	2×10^{-7}	3×10^{-6}	4×10^{-8}	1×10^{-7}	8×10^{-7}	0.004	2×10^{-16}	2×10^{-16}	2×10^{-16}	2×10^{-16}	2×10^{-16}
Obmed	1-2	-	-	-	-	-	-	-	-	-	-	-	-
	1-3	-	-	-	-	-	-	-	-	-	-	-	-
	1-4	-	-	-	-	-	-	-	-	-	-	-	-
	1-5	0.002	1×10^{-5}	1×10^{-4}	1×10^{-5}	2×10^{-5}	5×10^{-6}	9×10^{-5}	3×10^{-12}	3×10^{-11}	4×10^{-13}	4×10^{-13}	4×10^{-13}
	2-3	-	-	-	-	-	-	-	-	-	-	-	-
	2-4	-	-	-	-	-	-	-	-	-	-	-	-
	2-5	0.009	2×10^{-5}	8×10^{-5}	4×10^{-6}	4×10^{-6}	4×10^{-6}	5×10^{-4}	4×10^{-12}	6×10^{-12}	3×10^{-14}	2×10^{-14}	2×10^{-13}
	3-4	-	-	-	-	-	-	-	-	-	-	-	-
	3-5	-	2×10^{-5}	3×10^{-4}	3×10^{-5}	3×10^{-5}	5×10^{-5}	0.002	2×10^{-12}	4×10^{-11}	3×10^{-13}	2×10^{-13}	2×10^{-12}
	4-5	0.002	8×10^{-6}	5×10^{-5}	3×10^{-6}	3×10^{-6}	2×10^{-6}	5×10^{-5}	3×10^{-13}	2×10^{-12}	9×10^{-15}	8×10^{-15}	1×10^{-14}
OC	1-2	3×10^{-5}	-	-	-	-	-	1×10^{-9}	0.008	-	-	-	0.0118
	1-3	-	-	-	-	-	-	-	-	-	-	-	-
	1-4	2×10^{-8}	3×10^{-4}	-	-	-	0.021	3×10^{-13}	2×10^{-4}	-	-	-	0.0029
	1-5	5×10^{-13}	0.006	0.029	-	-	3×10^{-4}	2×10^{-16}	4×10^{-16}	6×10^{-15}	8×10^{-13}	2×10^{-10}	2×10^{-16}
	2-3	0.003	-	-	-	-	-	1×10^{-8}	0.015	-	-	-	0.0116
	2-4	-	0.02	-	-	-	-	-	-	-	-	-	-
	2-5	9×10^{-4}	-	0.037	-	0.031	0.007	8×10^{-15}	3×10^{-9}	8×10^{-13}	1×10^{-11}	1×10^{-10}	3×10^{-15}
	3-4	4×10^{-6}	0.005	-	-	-	-	2×10^{-12}	3×10^{-4}	-	-	-	0.0025
	3-5	2×10^{-10}	-	-	-	-	0.002	2×10^{-16}	2×10^{-16}	2×10^{-16}	2×10^{-14}	1×10^{-12}	2×10^{-16}
	4-5	-	-	-	-	-	-	1×10^{-10}	2×10^{-6}	5×10^{-11}	2×10^{-11}	1×10^{-10}	2×10^{-13}
midVermis	1-2	-	-	-	-	-	-						
	1-3	-	-	-	-	-	-						
	1-4	0.014	-	-	-	-	-						
	1-5	2×10^{-5}	2×10^{-4}	6×10^{-4}	0.005	0.009	2×10^{-4}						
	2-3	-	-	-	-	-	-						
	2-4	0.002	-	-	-	-	-						
	2-5	3×10^{-7}	8×10^{-9}	2×10^{-8}	6×10^{-7}	9×10^{-7}	6×10^{-9}						

	3-4	1×10^{-3}	-	-	-	-	-
	3-5	1×10^{-7}	4×10^{-8}	3×10^{-7}	4×10^{-6}	5×10^{-6}	3×10^{-8}
	4-5	0.047	1×10^{-7}	2×10^{-7}	4×10^{-6}	8×10^{-6}	6×10^{-6}
midOB	1-2	-	-	-	-	-	-
	1-3	-	-	-	-	-	-
	1-4	-	-	-	-	-	-
	1-5	0.036	1×10^{-4}	5×10^{-4}	7×10^{-5}	8×10^{-5}	8×10^{-5}
	2-3	-	-	-	-	-	-
	2-4	-	-	-	-	-	-
	2-5	-	1×10^{-4}	2×10^{-4}	2×10^{-5}	2×10^{-5}	4×10^{-5}
	3-4	-	-	-	-	-	-
	3-5	-	0.001	0.004	6×10^{-4}	5×10^{-4}	0.003
	4-5	-	2×10^{-4}	3×10^{-4}	3×10^{-5}	3×10^{-5}	7×10^{-5}

Box S1. Model Selection

```
Model selection based on AICc:

              K     AICc Delta_AICc AICcWt Cum.Wt        LL
NewGroup*VC 211 120790.1       0.00      1      1 -60180.24
NewGroup+VC  47 121666.2     876.08      0      1 -60785.90
NewGroup      6 122430.7    1640.59      0      1 -61209.34

Analysis of Variance Table

Model 1: full ~ NewGroup
Model 2: full ~ NewGroup + VC
Model 3: full ~ NewGroup * VC
  Res.Df      RSS  Df Sum of Sq       F    Pr(>F)
1  11965 19373292
2  11924 18049981  41   1323310 23.2679 < 2.2×10-16 ***
3  11760 16312786 164   1737195  7.6363 < 2.2×10-16 ***
---
Signif. codes:  0 ‘***’ 0.001 ‘**’ 0.01 ‘*’ 0.05 ‘.’ 0.1 ‘ ’ 1
```

AICc, Akaike Information Criterion corrected; Wt, weight; LL, log likelihood; Res, residuals; RSS, residual sums of squares; NewGroup, vaccine group; VC, virtual channel/source series for each ROI; full, full bandwidth power spectral density.
